# The magnetic resonance characteristics of sinonasal rhabdomyosarcoma in adults: analysis of 27 cases and comparison with pathological subtypes

**DOI:** 10.1186/s12880-023-01062-x

**Published:** 2023-07-28

**Authors:** Jun-hua Liu, Meng Qi, Wen-hu Huang, Yan Sha, Fang Zhang

**Affiliations:** Department of Radiology, Eye and ENT Hospital, Fudan University, No. 83 Fenyang Road, Xuhui District, Shanghai, 200031 China

**Keywords:** Magnetic resonance imaging, Nasal cavity, Paranasal sinus, Rhabdomyosarcoma, Adult

## Abstract

**Background:**

Sinonasal rhabdomyosarcoma (RMS) in adults is extremely rare, and early diagnosis and treatment are crucial to improve the patient’s prognosis. The purpose of this study was to evaluate the magnetic resonance imaging (MRI) findings of sinonasal RMS in adults and analyze the correlations between the imaging features and pathological subtypes.

**Methods:**

We reviewed 27 patients with pathologically proven RMS of the nasal cavity and paranasal sinuses, including embryonal RMS (ERMS) in 14 patients, alveolar RMS (ARMS) in seven patients, and mixed-type RMS in six patients. Conventional MRI was performed in all 27 patients, and high-resolution diffusion-weighted imaging was conducted in 25 patients. The tumor location, size, morphological features, signal intensity, texture, contrast enhancement characteristics, lymph node metastases, apparent diffusion coefficients (ADCs), and involvement of local soft tissues were independently assessed by two authors.

**Results:**

On MR imaging, sinonasal RMS appeared isointense on T1-weighted imaging in 21 cases (77.8%) and heterogeneously hyperintense on T2-weighted imaging in 18 patients (66.7%). After enhancement, the tumors were heterogeneously enhanced in 24 cases (88.9%). Botryoid enhancement with multiple small rings resembling bunches of grapes was found in 15 cases (55.6%). Mucosal invasion of the maxillary sinus was identified in 51.9% patients. Skull and orbit involvement were found in 55.6% and 81.5% patients, respectively. Lymph node metastasis was seen in 18 cases (66.7%). There were significant differences in botryoid enhancement (*P* = 0.044) and skull involvement (*P* = 0.044) among different histological subtypes. The mean ADC value of RMS was 0.73 ± 0.082 × 10^–3^ mm^2^/s, and there was no significant difference among different histological subtypes.

**Conclusions:**

Some characteristic MRI findings such as botryoid enhancement in the ethmoid sinus, involvement of the orbit and skull, and a lower ADC value can provide important clues for preoperative diagnosis of sinonasal RMS in adults. Further, botryoid enhancement was more common in ERMS, while skull involvement was more common in ARMS.

## Background

Rhabdomyosarcomas (RMS) are rare soft tissue malignancies that arise from myogenic cells and most commonly occur in children, representing more than 50% of all soft-tissue sarcomas in children [1, 2]. However, RMS less frequently occur in adults, accounting for only 3% of all adult soft-tissue sarcomas [3]. Although 40% of RMS arises in the head and neck region [4, 5], RMS originating in the paranasal sinuses is extremely rare, especially in adults [6, 7]. Due to its rarity and being prone to causing cranial nerve palsy and intracranial extension, sinonasal RMS often presents a major diagnostic and therapeutic challenge [8].

Early diagnosis and treatment are crucial to improve the patient’s prognosis. Magnetic resonance imaging (MRI) plays an important role in detecting sinonasal masses and in delineating the extent of disease because of the excellent tissue contrast it provides. However, information regarding imaging characteristics of sinonasal RMS in adults is very limited. Previous studies have either reported RMS data pooled with the head and neck RMS, or have focused on histological and clinical subsets of RMS [9–11]. Moreover, reports of MRI features of adult RMS in the sinonasal region are few [12, 13]. One recent study [14] analyzed MRI features of various pathological subtypes of sinonasal RMS. Unfortunately, the study just discussed conventional MRI features and the sample size of this case series is small.

In this article, we review MRI features including diffusion weighted imaging of the largest number of sinonasal RMS cases in adults to date in relation to their histological appearance to better understand the imaging features of this disease and facilitate earlier diagnosis.

## Methods

### Clinical data

**Fig. 1:**
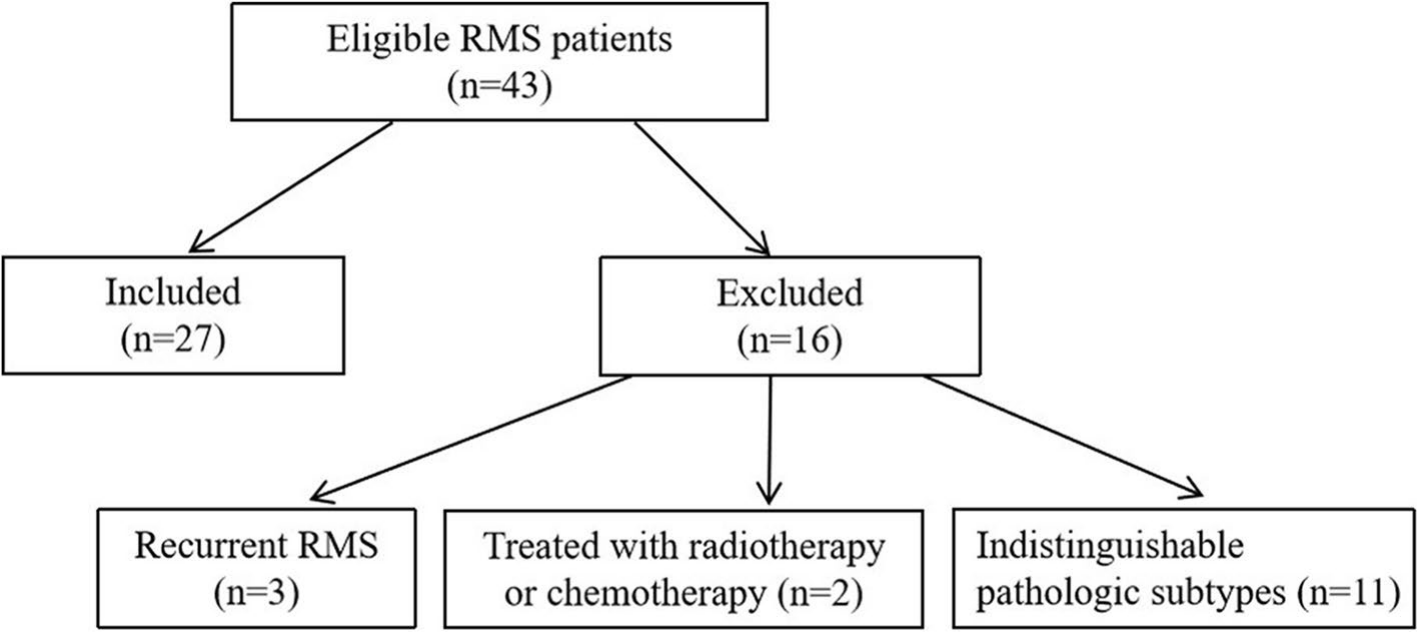
Flowchart of procedures performed in this study

This retrospective study was approved by the ethics committee of our hospital and the requirement for written informed consent was waived. We searched and identified patients with RMS of the nasal cavity and paranasal sinuses diagnosed by pathology in our hospital January 2013 and June 2021 from the database of our hospital. The inclusion criteria were: patients with RMS of the nasal cavity and paranasal sinuses who underwent sinonasal MRI examination within half a month before biopsy or surgery. Forty-three consecutive patients with RMS were collected. Of these 43 patients, three were excluded from the analysis because the tumors were recurrent, two were excluded because the patients were treated with radiotherapy or chemotherapy before MRI examination, and 11 cases were excluded due to patients with indistinguishable pathologic subtypes. The final study group was comprised of the remaining 27 patients, who underwent transnasal endoscopic biopsy or surgical resection at our institution (Fig. 1). The group consisted of 12 men and 15 women (age range, 18–65 years; mean age, 35.4 years). Histologic subtypes of the RMS included embryonal (ERMS) (*n* = 14), alveolar (ARMS) (*n* = 7), and mixed (embryonal/alveolar) type (*n* = 6). The clinical presentations, physical examination, surgery record, microscopic evaluation reports, and immunohistochemical data were extracted from the medical records. Strobe reporting guideline was used for this manuscript.

### MR Protocol

MRI was performed using a 3 T scanner (Magnetom Verio or Prisma; Siemens Medical, Erlangen, Germany) equipped with a 12-channel head coil for signal acquisition including conventional MRI in all patients and high-resolution diffusion-weighted imaging (RESOLVE-DWI) in 25 patients. The conventional nonenhanced MRI protocol used in this study included the following sequences: transverse T2-weighted fat-suppressed turbo spin-echo sequence (TSE, TR/TE = 4000/99 ms), coronal T2-weighted fat-suppressed turbo spin-echo sequence (TSE, TR/TE = 5500/103 ms), and transverse T1-weighted fast spin-echo sequence (TR/TE = 384/9.1 ms). The following parameters were used for the sequences: section thickness, 5 mm; intersection gap, 0.5 mm; matrix, 640 × 592; field of view (FOV), 220 × 220 mm.

RESOLVE-DWI was performed in the transverse plane using a readout-segmented echo-planar diffusion-weighted imaging sequence, and parallel imaging and 2D navigator-based reacquisition were used. The imaging parameters were as follows: TR/TE, 4700/66 ms; FOV, 240 × 240 mm; section thickness, 4 mm; intersection gap, 0.4 mm; number of excitations, 1; matrix, 192 × 192; gradient strength, b = 0, 1000 s/mm^2^. The data acquisition time was 2 min 15 s.

After the intravenous administration of Gd-DTPA (Magnevist, Bayer Schering, Berlin, Germany; 0.1–0.2 mmol/kg body), fat-suppressed T1-weighted Vibe images were obtained in transverse and coronal planes (TR/TE, 4.47/1.84 ms; thickness, 3 mm; intersection gap, 0.6 mm; matrix, 384 × 384; field of view, 230 × 230 mm).

### Imaging analysis

All the images were independently analyzed by two authors with 13 and 10 years of experience, respectively, in the field of head and neck diagnostic radiology. The tumor location, tumor extent (< 3 cavities or ≥ 3 cavities), margin (well-defined or ill-defined), shape (regular or irregular), signal intensity (hypointense, isointense, or hyperintense), and homogeneity (homogeneous or heterogeneous) on T1- and fat-suppressed T2-weighted images were evaluated. Homogeneity, degree of enhancement (mild, moderate, or marked), and botryoid enhancement (present or absent) on fat-suppressed contrast-enhanced T1-weighted image were also evaluated. Mucosal invasion of maxillary sinus, involvement of surrounding structures (skull or orbit), and lymph node metastases were determined.

The size of lesions was measured using the formula: lesion size = length (on transverse T2WI) × width (on transverse T2WI) × height (on coronal T2WI), where length, width, and height represented the maximum diameter. The ADC maps were generated automatically from RESOLVE-DWI (b = 0, 1000 s/mm^2^). The observers drew round regions of interest (ROIs) manually from all slices showing the lesion on the ADC images. The ROIs were carefully placed in the solid portion of the tumor, avoiding cystoid variation, hemorrhage, and necrosis. Then, the mean ADC value was selected as the final data.

### Histopathologic analysis

One senior pathologist with 10 years of experience in sinonasal pathological diagnosis, who was blinded to patient clinical records and MRI findings, evaluated histopathologic slides. The inspection reports of conventional microscopic hematoxylineosin staining and immunohistochemical data of all the lesions were collected.

### Statistical analysis

All statistical analyses were performed with SPSS 25.0 (IBM Corporation, Armonk, NY, USA) software. One-way analysis of variance (ANOVA) and chi-square test was used to test differences in ADCs and conventional MRI features among different histological subtypes. Kappa analysis was used to evaluate inter-reader reproducibility for the conventional MRI features. Intraclass correlation coefficient (ICC) was used to evaluate the intrareader reproducibility for ADCs. The ICC and kappa values were interpreted as follows: < 0.40, poor; 0.41–0.60, moderate; 0.61–0.80, good; and > 0.81, excellent. For all analyses, *P* < 0.05 was considered to indicate statistical significance.

## Results

### Patient characteristics

A total of 27 patients were included in this study. Patient age ranged from 18 to 65 years (mean, 35.4 years), and 48.1% (13/27) patients were aged from 20 to 40 years. Of these patients, 12 (44.4%) were male and 15 (55.6%) were female (Table 1). The most common presenting symptoms were nasal obstruction, headache, proptosis, diplopia, and cervical mass. The duration of medical history ranged from 2 weeks to 4 months.

**Table 1:** Demographic data of sinonasal RMS

	ERMS (*n* = 14)	ARMS (*n* = 7)	Mixed (*n* = 6)	Overall (27)
Gender				
Male	5(35.7%)	5(71.4%)	2(33.3%)	12(44.4%)
Female	9(64.3%)	2(28.6%)	4(66.7%)	15(55.6%)
Age at diagnosis				
Range	18–65	18–55	18–64	18–65
Mean	36.6 ± 16.3	33.1 ± 12.8	35.0 ± 17.4	35.4 ± 15.2
< 20	3(21.4%)	1(14.3%)	1(16.7%)	5(18.5%)
20–40	6(42.9%)	4(57.1%)	3(50.0%)	13(48.1%)
40–60	4(28.6%)	2(28.6%)	1(16.7%)	7(25.9%)
> 60	1(7.1%)	0(0%)	1(16.7%)	2(7.4%)

### MRI findings

Of the 27 RMSs, 23 (85.2%) were mainly located in the ethmoid sinus and/or nasal cavity region, two (7.4%) in the maxillary sinus, and two (7.4%) in the ethmoid and maxillary sinus region. One tumor (3.7%) was bilateral; the left side was involved in 12 tumors(44.4%) and the right side in 14 (51.9%). Conventional MRI features of RMS according to different subtypes and interobserver agreement between observers 1 and 2 are demonstrated in Table 2.

**Table 2:** Conventional MR imaging features of RMS

MR imaging features	RMS									
	ERMS		ARMS		Mixed		Overall		*P*	*k*
	N	%	N	%	N	%	N	%		
No. of patients	14	51.9	7	25.9	6	22.2	27	100		
Location									0.583	1.000
Ethmoid sinus and/or nasal cavity	12	85.7	7	100	4	66.6	23	85.2		
Maxillary sinus	1	7.15	0	0	1	16.7	2	7.4		
Ethmoid and maxillary sinus	1	7.15	0	0	1	16.7	2	7.4		
Tumor extent									0.842	0.697
< 3 cavities	3	21.4	2	28.6	2	33.3	7	25.9		
≥ 3 cavities	11	78.6	5	71.4	4	66.7	20	74.1		
Margin									0.617	0.649
Well-defined	1	7.1	0	0	0	0	1	3.7		
Ill-defined	13	92.9	7	100	6	100	26	96.3		
Shape									0.617	1.000
Regular	1	7.1	0	0	0	0	1	3.7		
Irregular	13	92.9	7	100	6	100	26	96.3		
Signal intensity on T1-weighted image									0.06	0.714
Hypointense	2	14.3	2	28.6	0	0	4	14.8		
Isointense	12	85.7	5	71.4	4	66.7	21	77.8		
Hyperintense	0	0	0	0	2	33.3	2	7.4		
Signal intensity on fat-suppressed T2-weighted image									0.585	0.727
Hypointense	0	0	0	0	0	0	0	0		
Isointense	5	35.7	3	42.9	1	16.7	9	33.3		
Hyperintense	9	64.3	4	57.1	5	83.3	18	66.7		
Homogeneity on T1-weighted image									0.72	0.707
Homogeneous	9	64.3	5	71.4	3	50	17	63		
Heterogeneous	5	35.7	2	28.6	3	50	10	37		
Homogeneity on fat-suppressed T2-weighted image									0.617	0.78
Homogeneous	1	7.1	1	14.3	0	0	2	7.4		
Heterogeneous	13	92.9	6	85.7	6	100	25	92.6		
Homogeneity on fat-suppressed contrast-enhanced T1-weighted image									0.617	0.649
Homogeneous	1	7.1	0	0	0	0	1	3.7		
Heterogeneous	13	92.9	7	100	6	100	26	96.3		
Degree of enhancement									0.092	0.75
Mild	1	7.1	0	0	2	33.3	3	11.1		
Moderate	5	35.7	3	42.9	4	66.7	12	44.45		
Marked	8	57.2	4	57.1	0	0	12	44.45		
Botryoid enhancement									0.044	0.773
Present	11	78.6	2	28.6	2	33.3	15	55.6		
Absent	3	21.4	5	71.4	4	66.7	12	44.4		
Mucosal invasion of maxillary sinus									0.383	0.851
Present	9	64.3	3	42.9	2	33.3	14	51.9		
Absent	5	35.7	4	57.1	4	66.7	13	48.1		
Skull involvement									0.044	0.777
Present	8	57.1	6	85.7	1	16.7	15	55.6		
Absent	6	42.9	1	14.3	5	83.3	12	44.4		
Orbit involvement									0.073	0.71
Present	13	92.9	6	85.7	3	50	22	81.5		
Absent	1	7.1	1	14.3	3	50	5	18.5		
Lymph node metastases									0.062	1.000
Present	12	85.7	4	57.1	2	33.3	18	66.7		
Absent	2	14.3	3	42.9	4	66.7	9	33.3		

**Fig. 2:**
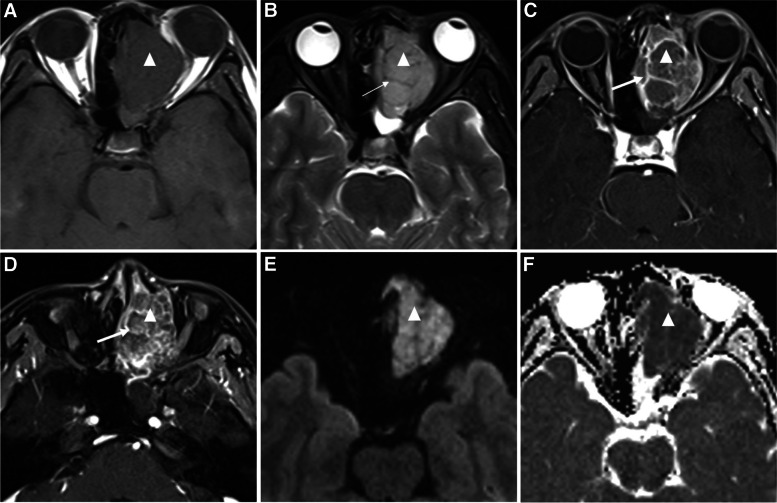
A 32-year-old female patient with ERMS. **a** T1-weighted, **b** T2-weighted, and **c**, **d** T1-weighted fat-suppressed postcontrast images show an irregular soft tissue mass (triangle) in the left ethmoid sinus along with involvement of the left orbit and sphenoid sinus. The size of the mass is 3.6 × 3.0 × 5.3 cm^3^ and the margin is ill-defined.The mass is isointense on T1WI and slightly hyperintense with low signal (thin arrow) on T2WI. After contrast enhancement, the mass is enhanced mildly and heterogeneously, and botryoid enhancement with multiple small rings (thick arrow) can be seen. **e** DWI shows high signal in the mass (triangle). **f** ADC map shows the evident low signal (triangle) with a measured ADC of 0.649 × 10^–3^ mm^2^/s

**Fig. 3:**
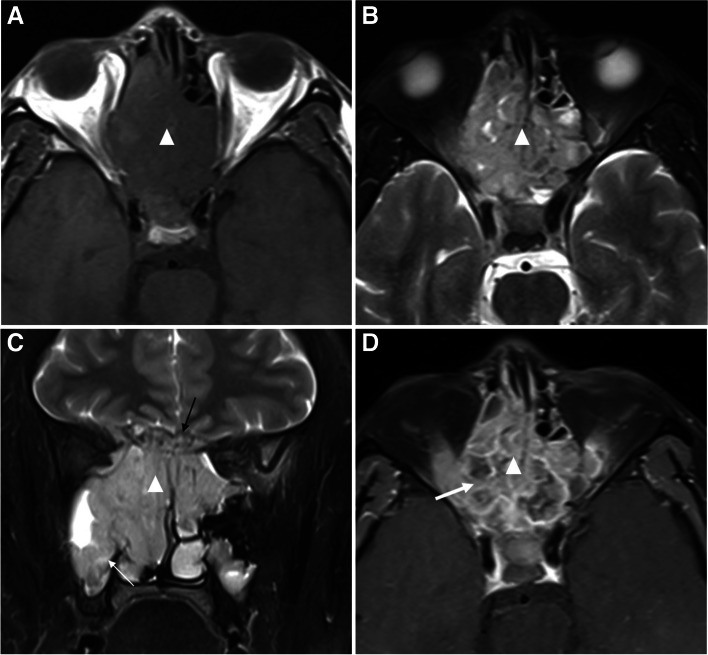
A 36-year-old male patient with ERMS. **a** T1-weighted, **b**, **c** T2-weighted, and **d** T1-weighted fat-suppressed postcontrast images show an irregular soft tissue mass (triangle) in both ethmoid sinuses. The size of the mass is 5.1 × 5.1 × 5.4 cm^3^ and the margin is ill-defined. Mucosal invasion of maxillary sinus (thin white arrow) and skull involvement (thin black arrow) can be seen. After contrast enhancement, the mass is enhanced moderately and heterogeneously, and botryoid enhancement with multiple small rings (thick arrow) can be seen

**Fig. 4:**
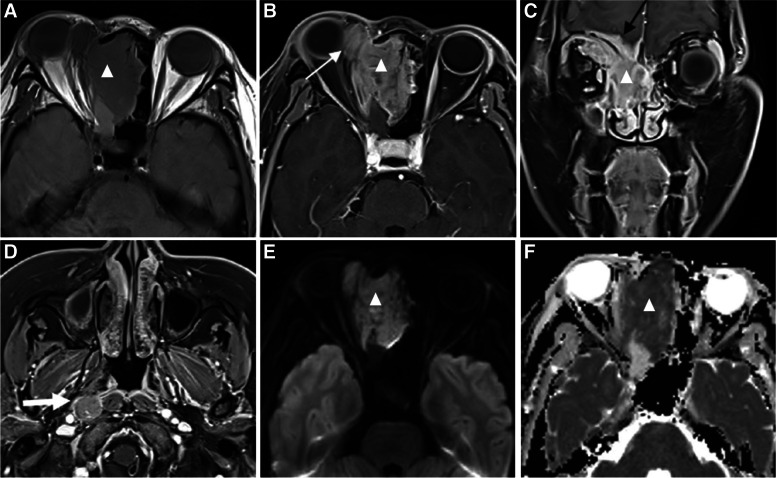
A 39-year-old female patient with ARMS. **a** T1-weighted and **b**, **c**, **d** T1-weighted fat-suppressed postcontrast images show an irregular soft tissue mass (triangle) in the right ethmoid sinuses with right retropharyngeal lymph node metastasis (thick white arrow). The size of the mass is 4.6 × 3.6 × 4.2 cm^3^ and the margin is ill-defined. After contrast enhancement, the mass is enhanced markedly and heterogeneously. Orbit involvement (thin white arrow) and skull involvement (thin black arrow) can be seen. **e** DWI shows slightly high signal in the mass (triangle). **f** ADC map shows a low signal (triangle) with measured ADC of 0.863 × 10^–3^ mm^2^/s

The mean size of sinonasal RMS are presented in Table 3, and there was no significant difference among different histological subtypes.The mean size of the RMS was 4.4 × 3.3 × 4.7 cm^3^. Further, 96.3% (26/27) of the masses had ill-defined borders and irregular shapes, and multi-cavity growth (cavities, n ≥ 3) was identified in 74.1% (20/27) cases. Compared with gray matter, MRI showed that 21 cases (77.8%) were isointense, four cases were slightly hypointense and two cases were slightly hyperintense on T1WI. Nine cases were isointense and 18 cases (66.7%) were hyperintense on T2WI. Furthermore, 92.6% (25/27) cases were heterogeneous on T2WI, with small areas of cystic, necrotic, or low signal (Fig. 2). After contrast enhancement, 96.3% (26/27) masses were heterogeneously enhanced (Figs. 2, 3 and 4), and botryoid enhancement with multiple small rings resembling bunches of grapes was found in 15 cases (55.6%) (Figs. 2 and 3), including 11 ERMS, two ARMS, and two mixed-type RMS. Mucosal invasion of maxillary sinus was identified in 51.9% (14/27) patients (Fig. 3). Skull and orbit involvement were noted in 55.6% (15/27) and 81.5% (22/27) patients, respectively (Figs. 2, 3 and 4). Lymph node metastasis was seen in 18 cases (66.7%), including five cases of retropharyngeal lymph node metastasis (Fig. 4), nine cases of cervical lymph node metastasis, and four cases of both retropharyngeal lymph node and cervical lymph node metastasis.

**Table 3:** Mean size of the sinonasal RMS

	ERMS (*n* = 14)	ARMS (*n* = 7)	Mixed (*n* = 6)	Overall (*n* = 27)
Mean size(cm^3^)	4.4 × 3.6 × 5.0	4.4 × 2.9 × 4.4	4.4 × 3.2 × 4.5	4.4 × 3.3 × 4.7

Of all the above imaging features, significant differences were found in botryoid enhancement (*P* = 0.044) and skull involvement (*P* = 0.044) among different histological subtypes. Good interobserver agreement was achieved for botryoid enhancement (*k* = 0.773) and skull involvement (*k* = 0.777). Botryoid enhancement was more likely in ERMS than ARMS and mixed type, while skull involvement was more likely in ARMS than ERMS and mixed type. The ADCs of sinonasal RMS and ICC are presented in Table 4. The mean ADC value of RMS was 0.73 ± 0.082 × 10^–3^ mm^2^/s, and there was no significant difference among different histological subtypes.

**Table 4:** Mean ADCs of sinonasal RMS

	ERMS	ARMS	Mixed	*P*	ICC
Value of ADCs (× 10^–3^ mm^2^/s)	0.72 ± 0.07	0.759 ± 0.077	0.719 ± 0.115	0.323	0.84

## Discussion

RMS is an aggressively malignant neoplasm, derived from the mesenchymal cells that arise anywhere in the body [15]. The current WHO classification (World Health Organization; WHO 2020) [16] divides RMS into four subtypes: embryonal, alveolar, pleomorphic, and spindle cell/sclerosing RMS. The embryonal type is the most common type and mainly affects children; whereas, pleomorphic RMS is the predominant type in adults and accounts for > 50% of all adult RMS and increases in incidence with age [17]. A previous study reported that alveolar type RMS is the most common type in adults in the head and neck region [18]. However, the present study exhibited an embryonal type predominance (51.9%) of sinonasal RMS in adults, wherein only 25.9% cases had alveolar type RMS and none had the pleomorphic and spindle cell/sclerosing type. This difference can be attributed to certain factors. Mixed (embryonal/alveolar) type RMS was included in this study, which maybe affect the proportion of different types of RMS; some not otherwise specified RMS were excluded from the study.

Sinonasal RMS poses a significant risk for subarachnoid dissemination and is often unresectable by the time it is diagnosed. Early diagnosis and management are therefore crucial to patients’ survival. MRI is the best imaging modality for assessment of RMS, and it has been reported to be highly effective in precisely determining the site of tumor origin, local spread, and regional nodal involvement [9]. However, few studies in the literature have characterized MRI features of sinonasal RMS in adults. One recent study [13] evaluated and compared the multiparametric MRI findings of sinonasal RMS in adults and carcinoma and found that using conventional MRI with added ADCs had the potential to improve the diagnostic accuracy of adult sinonasal RMS. However, the study mainly compared the difference between RMS and carcinoma. Moreover, the RMS patient sample size was relatively small. Our study included the largest number of adult sinonasal RMS cases to date and compared MRI features of sinonasal RMS in adults among different RMS subtypes.

Based on our results, most RMS in the sinonasal region appeared isointense on T1WI, heterogeneously hyperintense on T2WI, and showed inhomogeneous moderate-to-marked enhancement. These conventional imaging findings are consistent with that of adult RMS in other parts of the body [18]. In our study, botryoid enhancement pattern was found in 55.6% (15/27) patients, and all the cases were located in the ethmoid sinus which is similar to a previous study [9]. However, our results do not support the previous view which stated that botryoid enhancement indicated the botryoid subtype of ERMS in which mucoid-rich stroma is covered with a thin layer of tumour cells [9, 19]. In our study, although 73.3% of botryoid enhancement pattern occurred in ERMS, there were still two cases each of the ARMS and mixed RMS type. In addition, the results demonstrated significant differences in botryoid enhancement among different RMS subtypes, which suggested that botryoid enhancement was more likely in ERMS than ARMS and mixed-type RMS.

RMS is an aggressive malignancy that can spread extensively via direct extension. In our study, the ethmoid sinus and/or nasal cavity were the most common sites, but multi-cavity growth (≥ 3 cavities) was demonstrated in 74.1% patients. An interesting observation in this study was that the invasion of maxillary sinus was along the mucous membrane, rather than directly into the maxillary sinus cavity, which was different from that in carcinoma. Another characteristic feature was the involvement of the orbit and skull. In the current study, orbital invasion (81.5%) was more common than skull invasion (55.6%). The results revealed significant differences in skull involvement among the different RMS subtypes. ARMS was more likely to invade the skull than ERMS and mixed type, which also supported the opinion that most ARMS are more aggressive than ERMS. Lymph node metastases is an important route for RMS progression. In the present study, the frequency of lymphatic metastasis was 66.7%, higher than that reported in a previous study with 20% lymph node metastasis(2/10) [9].

DWI has been recognized as an important tool to improve the diagnosis of sinonasal tumors [13, 20]. A recent study [13] indicated that the ADCs of sinonasal RMS were significantly lower than those of sinonasal carcinomas. Therefore, we attempted to compare the ADC values of different types of RMS. However, no significant difference was found in ADCs among different histological subtypes. In our study, the mean ADC value of RMS was lower than that reported in a recent study with the mean ADC value of 0.992 ± 0.133 × 10^–3^ mm^2^/s [13]. The difference may be related to different machines and measuring methods.

Our study has some limitations. First, the sample size of ARMS and mixed-type RMS was relatively small. Second, statistical analysis of different RMS subtypes was not adequate to distinguish the histological subtypes. Therefore, further artificial intelligence-based research such as radiomics or texture analysis is required in future studies.

## Conclusions

In conclusion, our study indicated that botryoid enhancement in the ethmoid sinus, mucosal invasion of the maxillary sinus, involvement of the orbit and skull, lymph node metastases, and a lower ADC value are likely the characteristic MRI features of sinonasal RMS in adults. Further, botryoid enhancement was more common in ERMS, while skull involvement was more common in ARMS.

## Data Availability

The datasets used and analysed during the current study are available from the corresponding author on reasonable request.

## Abbreviations

RMS: Rhabdomyosarcomas
ERMS: Embryonal rhabdomyosarcomas
ARMS: Alveolar rhabdomyosarcomas
RESOLVE-DWI: High-resolution diffusion-weighted imaging
ADC: Apparent diffusion coefficient
